# The Nagoya Protocol on access and benefit sharing: The neglected issue of animal health

**DOI:** 10.3389/fmicb.2023.1124120

**Published:** 2023-02-14

**Authors:** Maria Serena Beato, Valentina Veneroso

**Affiliations:** ^1^National Reference Laboratory for African Swine Fever, Istituto Zooprofilattico Sperimentale dell'Umbria e delle Marche (IZSUM), Perugia, PG, Italy; ^2^Independent Researcher, Udine, Italy

**Keywords:** Nagoya Protocol, animal health, pathogens, access and benefit sharing, zoonosis

## 1. Introduction

**Figure 1:**
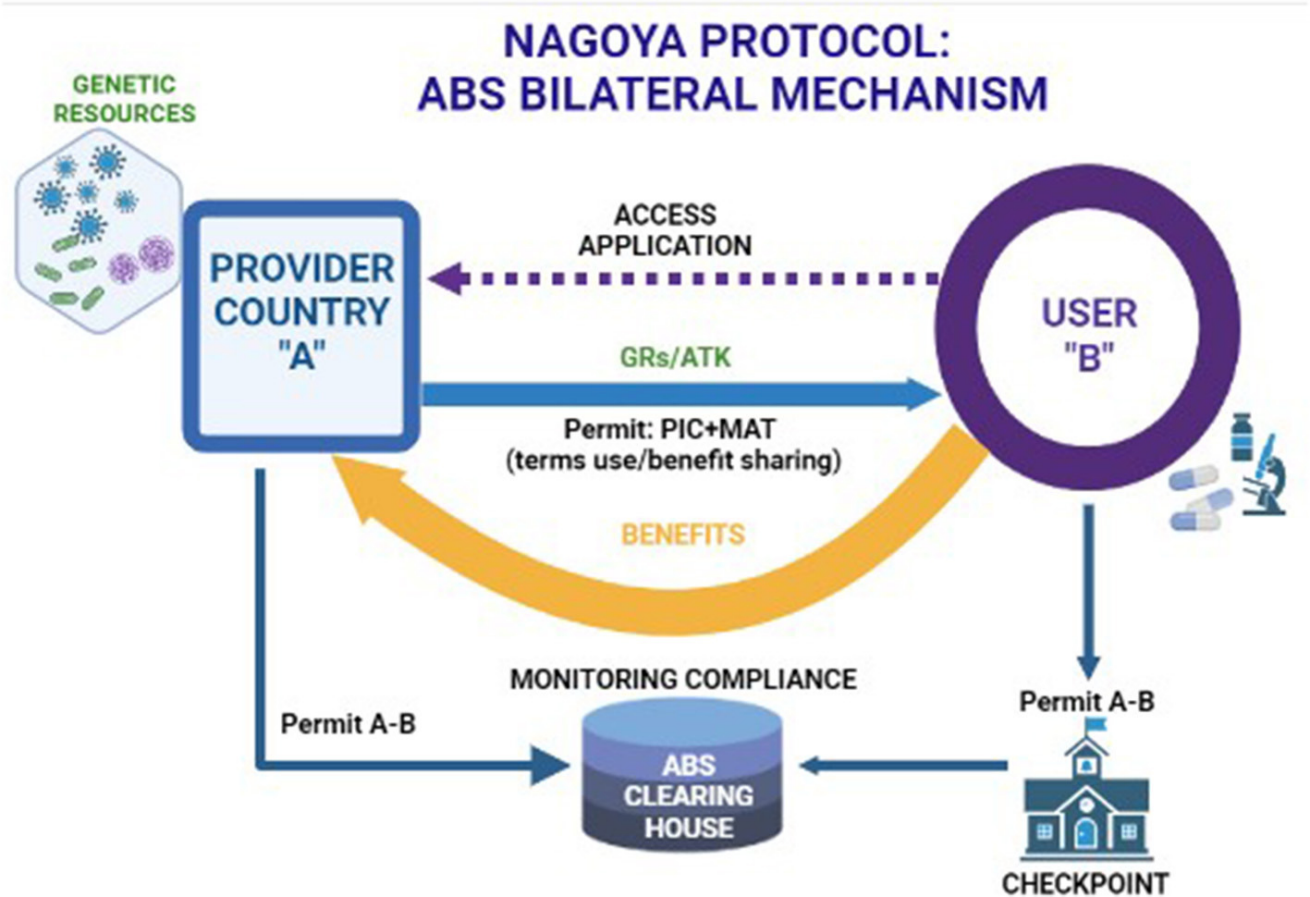
The ABS bilateral mechanism of the Nagoya Protocol. Created in Biorender.com accessed on 28th December 2022.

On 12 October 2014, the Nagoya Protocol (NP) to the Convention on Biological Diversity (CBD) entered into force. The protocol sets an international legally binding framework for the access to genetic resources (GRs) and the fair and equitable sharing of benefits arising from their utilization, conceived as the third objective of the CBD, and known as access and benefit sharing (ABS). The major aim of the ABS system, shaped by the CBD and the NP, is to fight biopiracy and restore fairness and equity in the exchange of GRs and rests on two pillars. The first pillar is grounded on the acknowledgment of sovereign rights of countries over their natural resources, a principle affirmed for the first time in the Convention of Biological Diversity (1992) (NP art. 6par. 1; art. 15 par. 1 CBD) (Convention of Biological Diversity, 1992; Nagoya Protocol, 2010). This principle encompasses the right and the authority of each country to govern the access to GRs within their territory, controlling and monitoring their use. The second pillar is grounded on the users' compliance with ABS rules: GR and associated traditional knowledge (ATK) users shall utilize them only upon “legal” access (art.15 NP) (Nagoya Protocol, 2010). More specifically, where the provider country has adopted ABS domestic measures, users shall seek an express authorization for access, Prior Informed Consent (PIC) and shall share the benefits arising from their utilization with that country “in a fair and equitable way,” based on the binding agreement: Mutually Agreed Terms (MATs) (art. 5 NP) (Figure 1) (Nagoya Protocol, 2010). Benefits shared should be directed toward the conservation of biological diversity and the sustainable use of its components (art. 9 NP) (Nagoya Protocol, 2010). The implementation of the NP has been posing a number of challenges that emerged with the operationalization of ABS principles. Such a process involves different actors and rules, allocated in a complex layered structure that spans from the global to the national level and to a contractual level between the user and the provider country (Lajaunie and Morand, 2020). Users have to deal with a wide variety of domestic ABS measures: which means that they shall engage in a treacherous “case-by-case” due diligence before using GRs, in order to ascertain whether their specific activities fall in the scope of such national rules. Subsequently, users shall enter an onerous and often time-consuming process with the ABS competent authorities to obtain a PIC and negotiate a MAT. Amplified by the complexity of such a multilevel governance framework, the most insidious challenge of NP is posed by the extremely broad scope of the treaty, based on the definition of keywords such as “genetic resource” and “utilization.” The NP, indeed, applies to all non-human GRs meant as any genetic “material of actual or potential value of plant, animal and microbial origin,” when they are “utilized,” that is “to conduct research and development” on their “genetic and/or biochemical composition.” Each country, within the vast perimeter of such definitions, may decide to shape the scope of their domestic ABS measures in the most extensive or restrictive way. For instance, each country may or may not include certain GRs and even the related digital sequence information, therefore stretching the term “utilization” up to encompass basic research unlinked to any further potential development or certain “upstream” activities on GRs such as their mere description and identification. While most of these challenges concern a broad range of scientific and economic fields, the aim of this article was to provide an overview of specific issues regarding the application of NP in the animal health sector.

### 1.1. One size does not fit all: Nagoya Protocol and pathogens

Pathogens and pathogenic material certainly meet the formal definition of GR set by the NP. Given their potential to become a threat to global health, their subjection to the NP ABS bilateral system surfaced as a cause of concern during its negotiation. Preparedness and response actions to emerging or re-emerging global public health threats rely on prompt access to such GRs: case-by-case ABS negotiations with national competent authorities could hamper this goal. Furthermore, pathogens do not respect borders and as demonstrated by the COVID-19 pandemic, they easily spread in a globalized world. To acknowledge the necessity to safeguard prompt access to pathogens, the final draft of NP included article 8.(b), which claimed “special consideration” for pathogens and pathogenic material. This provision requires parties to pay “due regard to cases of present or imminent emergencies that threaten or damage human, animal or plant health, as determined nationally or internationally.” The protocol further establishes that in such cases, “Parties may take into consideration the need for expeditious access to genetic resources and expeditious fair and equitable sharing of benefits arising out of the use of such genetic resources, including access to affordable treatments by those in need, especially in developing countries' (Wilke, 2013; Morgera et al., 2015).” Indeed, the issue of the suitability of this bilateral ABS regime to pathogens maybe “given their seemingly doubtful status as objects worthy of environmental conservation efforts” (Rourke, 2020), had never been at the stake of the debate until the final phases of the NP negotiation. In fact, such a topic emerged for the first time triggered by the avian influenza (AI) H5N1 international crisis. In particular, in 2007, Indonesia refused to share AI-positive samples with WHO based on their sovereignty over the virus isolated within their territory (Garrett and Fidler, 2007). Indonesia claimed that – as per CBD principles – access to the GRs should have been subjected to PIC and the ABS mechanism should have been negotiated in a specific agreement. To solve this dilemma, the WHO negotiated the Pandemic Influenza Preparedness (PIP) Framework, adopted as a non-binding resolution in 2011, to improve and strengthen the sharing of “H5N1 and influenza viruses with human pandemic potential” and ensure a “transparent, equitable, efficient, effective system for [...] access to vaccines and sharing of other benefits” (article 2 PIP Framework). This resolution was conceived as an alternative ABS mechanism more suitable to the case, but still consistent with the CBD principles. The CBD parties, meanwhile, strove to find an acceptable compromise regarding the inclusion of pathogens as developed countries pursued the exclusion of pathogens or “specific uses of pathogens” from NP's scope (CBD Ad Hoc Open Ended Working Group on Access and Benefit Sharing, 2009), and developing countries stood firm for their broad inclusion. The call for special consideration finally adopted in article 8(b) was adopted in 2010 appearing as a weak and perhaps hasty solution, rather than an appropriate regulation of such a sensitive issue. Although the scope of article 8(b) is reasonably broad, covering all kinds of health emergencies for humans, animals, and plants and all related GRs, substantial discretion is left to each country on how to implement such measures at a national level potentially jeopardizing the laudable ratio of this provision. The provision, again, relies on the subjective interpretation of each NP party of wide and undefined concepts such as “pay due regard”, “present or imminent emergencies,” and “expeditious access.” This concern appears to be confirmed by data provided in the 2018 analysis of interim national reports on ABS published by the Subsidiary Body on Implementation of the NP. In fact, only 56% of NP parties reported paying attention to the cases of present or imminent emergencies that threaten or damage health as per article 8(b) in the implementation of their ABS measures. In contrast, only a minority considered the need for expeditious access and benefit-sharing procedures (CBD Executive Secretary, 2018). Moreover, conflicting incidents based on sovereignty on viruses claims occurred again in recent years (i.e., 2012 – MERS-CoV - Saudi Arabia; 2014–2016 Ebola Crisis – West Africa) (Rourke, 2020).

### 1.2. The animal health sector and the Nagoya Protocol

The COVID-19 pandemic has once again raised the issue of how NP may hinder the fast and predictable access and sharing of pathogens and related information to face a global human public health threat (DiEuliis, 2015), debated even before the pandemic (Sett et al., 2022). In contrast, the impact of the ABS system shaped by NP on the animal health sector still appears as a rather neglected issue.

Many activities usually conducted for monitoring and control purposes in the animal health sector might be considered as a “utilization” within the scope of the NP, exposing such practices to the ABS negotiation process. This is the case, for example, of the sequencing of an animal pathogen, necessary to classify the disease it causes, as notifiable or not, to animal health authorities. Some NP parties, under their ABS domestic measures, may address GR characterization and sequencing activities as “research” or “utilization” triggering benefit-sharing obligations (i.e., Malawi, India CBD Ad Hoc Technical Expert Group on Digital Sequence Information on Genetic Resources, 2020). In contrast, in the EU, the “taxonomic identification of biological or genetic material, by morphological or molecular analysis, including through the use of DNA sequencing” is to be considered to “precede utilization,” therefore not triggering compliance obligations on ABS for the user (European Commission, 2016).

The lack of clarity and common understanding among NP parties of the NP core concept of “utilization” is amplified by the unavailability of internationally agreed definitions for the animal health sector of “research” and – for instance – “diagnosis,” which could be used as valuable reference tools for lawmakers and operators to address the issue. In the context of such ambiguity, the research on animal microorganisms that may have an impact in predicting and preventing emerging and re-emerging threats for animals and humans might be severely affected, as well as the activities of international reference laboratories (RLs) itself. With regard to this latter issue, the institutional mission of designated RLs is to provide scientific and technical assistance and expert advice on diagnosis and control of the disease/pathogen. To pursue this goal, RLs receive, store, and analyze pathogens and pathogenic material received from foreign countries for disease confirmation and the development of diagnostic tools. In this contest, RLs have the mandates to assist member countries of the network in the diagnosis of a given animal disease, characterization of the disease agent and pathogen, and development of diagnostic tools in order to optimize and implement improved detection for improved surveillance. The lack of clarity of the NP, in its current form, poses challenges for RLs in executing their mandates strictly linked to the overall goal of combating animal diseases that have an impact on animal health at national and potentially international levels. The call for “special consideration” for “human, animal or plant health” provided by NP article 8(b) does not appear an adequate response to such concerns. With regard to the animal health sector, a common international understanding of the “cases of present or imminent emergencies that threaten or damage animal [...] health” that, according to article 8(b), should trigger expeditious access to GRs and sharing of benefits arising out of the use of such GRs is not available. At a first glance, no definition of “emergency” has been ever issued by international animal health organizations.

To date, the only internationally accepted definition of “emergency,” recalled by NP, has been provided for the human public health sector by WHO in the International Health Regulation (International Health Regulation, 2022) as “an extraordinary event which is determined to constitute a public health risk to other States through the international spread of disease and to potentially require a coordinated international response.” However, this definition would be rather restrictive for the animal health sector and could not easily be applied mutatis mutandis: not necessarily, indeed, an animal health emergency constitutes a public health risk, unless a zoonotic pathogen is involved.

## 2. Discussion

The WHO stated that, over the last decade, microorganisms from animals or animal products have caused approximately 75% of the new diseases in humans. This highlights the importance of investigating, with a preventive approach, the role of the animal reservoir in maintaining and spreading such microorganisms that may or may not act as pathogens in animals but may have the potential to cause disease and pandemics in humans. In this view, the research in veterinary microbiology represents an essential piece for the prediction and prevention of emerging and re-emerging diseases in the One-Health approach that does not find sufficient recognition in the NP, neglecting the basic research on animal microorganisms and impairing the capacity to fight epidemics globally. The ambitious idea of an effective, consistent, and user-friendly worldwide implementation of the ABS system shaped by the NP is hampered by the extremely broad scope of the treaty. The definitions of the keywords appear to be a core element for GR users that shall apply due diligence. Where such definitions are provided or suggested, they explicitly apply in a restricted context and may not be adopted in others, in particular bringing to light the forgotten and underestimated role of animal health in health emergencies and its impact on the implementation of ABS worldwide. Surely, awareness of the difficulties in applying the ABS system for the animal health sector should arise in a timely manner bringing the debate to the attention of local/regional, national, and international stakeholders. A global call to action of animal health specialists in raising the issue locally and globally is necessary.

## 3. Conclusion

In conclusion, there is a need for fast action of international and national animal health organizations and institutions in putting this at the top of their agenda, creating a new ABS mechanism for animal GRs, namely, animal microorganisms, alternative to the bilateral system of NP, in which access is facilitated through standardized global rules for benefit-sharing, with an eye to ABS multilateral mechanisms developed in other international fora.

## Author contributions

All authors listed have made a substantial, direct, and intellectual contribution to the work and approved it for publication.
